# Somatic Variants in the Human Lens Epithelium: A Preliminary Assessment

**DOI:** 10.1167/iovs.16-19726

**Published:** 2016-08-08

**Authors:** Rosana Mesa, Manoj Tyagi, George Harocopos, David Vollman, Steven Bassnett

**Affiliations:** 1 Department of Ophthalmology and Visual Sciences, Washington University School of Medicine, St. Louis, Missouri, United States; 2 Genome Technology Access Center, Department of Genetics, Washington University School of Medicine, St. Louis, Missouri, United States; 3 Department of Pathology and Immunology, Washington University School of Medicine, St. Louis, Missouri, United States

**Keywords:** somatic mutation, cataract risk factors, genomics

## Abstract

**Purpose:**

We hypothesize that somatic mutations accumulate in cells of the human lens and may contribute to the development of cortical or posterior sub-capsular cataracts. Here, we used a Next-generation sequencing (NGS) strategy to screen for low-allelic frequency variants in DNA extracted from human lens epithelial samples.

**Methods:**

Next-Generation sequencing of 151 cancer-related genes (WUCaMP2 panel) was performed on DNA extracted from post-mortem or surgical specimens obtained from 24 individuals. Usually, pairwise comparisons were made between two or more ocular samples from the same individual, allowing putative somatic variants detected in lens samples to be differentiated from germline variants.

**Results:**

Use of a targeted hybridization approach enabled high sequence coverage (>1000-fold) of the WUCaMP2 genes. In addition to high-frequency variants (corresponding to homozygous or heterozygous SNPs and Indels), somatic variants with allelic frequencies of 1-4% were detected in the lens epithelial samples. The presence of one such variant, a T > C point substitution at position 32907082 in *BRCA2*, was verified subsequently using droplet digital PCR.

**Conclusions:**

Low-allelic fraction variants are present in the human lens epithelium, at frequencies consistent with the presence of millimeter-sized clones.

Clouding of the ocular lens, or cataract, is the leading cause of blindness globally, affecting tens of millions of people.^1^ Cataracts are classified according to where in the lens opacities are located. Nuclear cataracts, for example, involve the center of the lens, whereas cortical cataracts are located in the periphery. Posterior subcapsular cataracts (PSCs), affect a layer of cells immediately beneath the posterior lens pole. Although loss of tissue transparency is the common endpoint, each type of cataract affects a particular region of the lens, is associated with a unique spectrum of risk factors, and probably reflects a distinct etiology.^2^

Work from many laboratories supports the notion that the progressive aggregation of aged and extensively modified lens proteins is the proximate cause of nuclear cataracts (reviewed in Ref. 3). The etiology of cortical cataracts and PSCs is less well established, even though, collectively, these subtypes can account for most cataracts in some populations.^4^ Several observations support the idea that the accumulation of somatic mutations in the nucleated cells of the lens could play a role in the development of cortical cataracts and PSCs, at least under certain conditions. The strongest evidence comes from longitudinal studies of human cohorts exposed to ionizing radiation. The lens is among the most radiation-sensitive tissues in the body, second perhaps only to the germ cells.^5^ In astronauts,^6^ Chernobyl cleanup workers,^7^ radiologic technicians,^8^ and patients exposed to radiotherapy,^9^ studies have consistently identified an association between radiation exposure and latent development of cataracts. It should be noted, however, that it is notoriously difficult to collect accurate dosimetry data in long-term retrospective studies,^10^ and this caveat should be borne in mind when considering the conclusions of such studies. One cohort that has been carefully followed for many decades consists of survivors from the atomic bomb attacks on Hiroshima and Nagasaki. Careful analysis of cataract in atomic bomb survivors has shown that both the prevalence and incidence of cataracts (generally cortical cataracts and PSCs) that required surgical removal are increased at doses of 0.5 Gy or lower.^11,12^ Those who were young at the time of exposure appear to have been at particular risk. These and other findings have led the International Commission on Radiological Protection to recently recommend lowering the exposure threshold for vision-impairing cataract from 5 Gy to 0.5 Gy.^5^

A second line of evidence comes from epidemiological studies that have examined the link between cortical cataract formation and lifetime UV-B exposure. Ultraviolet-B radiation is a potent environmental mutagen of particular relevance to the eye. Due to their relatively high energy, UV-B photons may be absorbed directly by double bonds in pyrimidine bases (thymine and cytosine) promoting the formation of cyclobutane pyrimidine dimers (CPDs) and 6-4 photoproducts in the DNA; two helix-distorting photo-lesions. Photo-lesions are usually repaired quickly through the nucleotide excision repair pathway, but unrepaired adducts can cause mispairing during DNA replication, leading to the formation of classic UV signature mutations C > T and CC > TT.^13^ In animal models, UV-B (at levels equivalent to ambient exposure) can penetrate the cornea, leading to the production of CPDs and 6-4 photoproducts in the DNA of anterior lens epithelial cells.^14^ In humans, most epidemiological studies examining risk factors for cataract have concluded that exposure to UV radiation (and, in particular, UV-B) is associated with an increased risk of cortical cataract.^15^ Based on such data, the World Health Organization estimates that, globally, 20% of cataracts may be attributed to UV exposure. It is important to acknowledge, however, that many of the detrimental effects of UV exposure (particularly acute exposure) to the lens involve nonmutagenic mechanisms (such as type I photo-oxidation reactions^16^).

If somatic mutations indeed play a contributory role in cataract formation, one might expect that defects in DNA repair pathways, which typically result in increased mutational frequency,^17^ would be associated with an elevated risk of cataract. In keeping with this notion, cataract is a common feature of several syndromic conditions caused by mutations in DNA-repair genes, including Cockayne syndrome, Werner syndrome, Rothmund-Thompson syndrome, and Trichothiodystrophy.^18^ Similarly, polymorphisms in *XRCC1* (base excision repair pathway) and *XPD* (nucleotide excision repair pathway) are associated with the development of age-related cataract.^19^

Somatic variants, should they occur in the human lens epithelium, are likely to be present at low frequencies, making it technically challenging to detect them against the large background signal from the wild-type genome. In this study, therefore, we elected to use a targeted hybridization next-generation sequencing (NGS) strategy to screen a panel of 151 genes for the presence of somatic variants. By focusing on a restricted gene set, we were able to achieve sufficient depth of coverage to allow the detection of variants present at variant allele frequencies as low as 1%. Our data suggest that somatic variants are present in the human lens epithelium, at frequencies consistent with the presence of millimeter sized clones. The potential implications of this finding for cataract formation are discussed in relation to the clonal organization of the lens epithelium and the lifelong growth process of the lens.

## Materials and Methods

### Lens Epithelial Samples

Intact, de-identified human eyes or isolated lenses were obtained from Mid-America Transplant Services (St. Louis, MO, USA), Saving Sight (Kansas City, MO, USA), and the autopsy service of the Department of Pathology and Immunology (Washington University, St. Louis, MO, USA). Samples were obtained less than 48 hours postmortem and dissected immediately on arrival at the laboratory. In addition to donor lenses, surgical capsulorhexis specimens also were used. Capsulorhexis samples are small flaps of central anterior lens capsule with adherent epithelial cells and are removed (and routinely discarded) in the course of extracapsular cataract surgery. Ethical approval for the capsulorhexis study was obtained from the Washington University Human Research Protection Office (HRPO), and written informed consent was provided by all participants before enrollment, in accordance with the tenets of the Declaration of Helsinki and Health Insurance Portability and Accountability Act (HIPAA) regulations. A description of the samples used in this study is provided in Table 1.

**Table 1:**
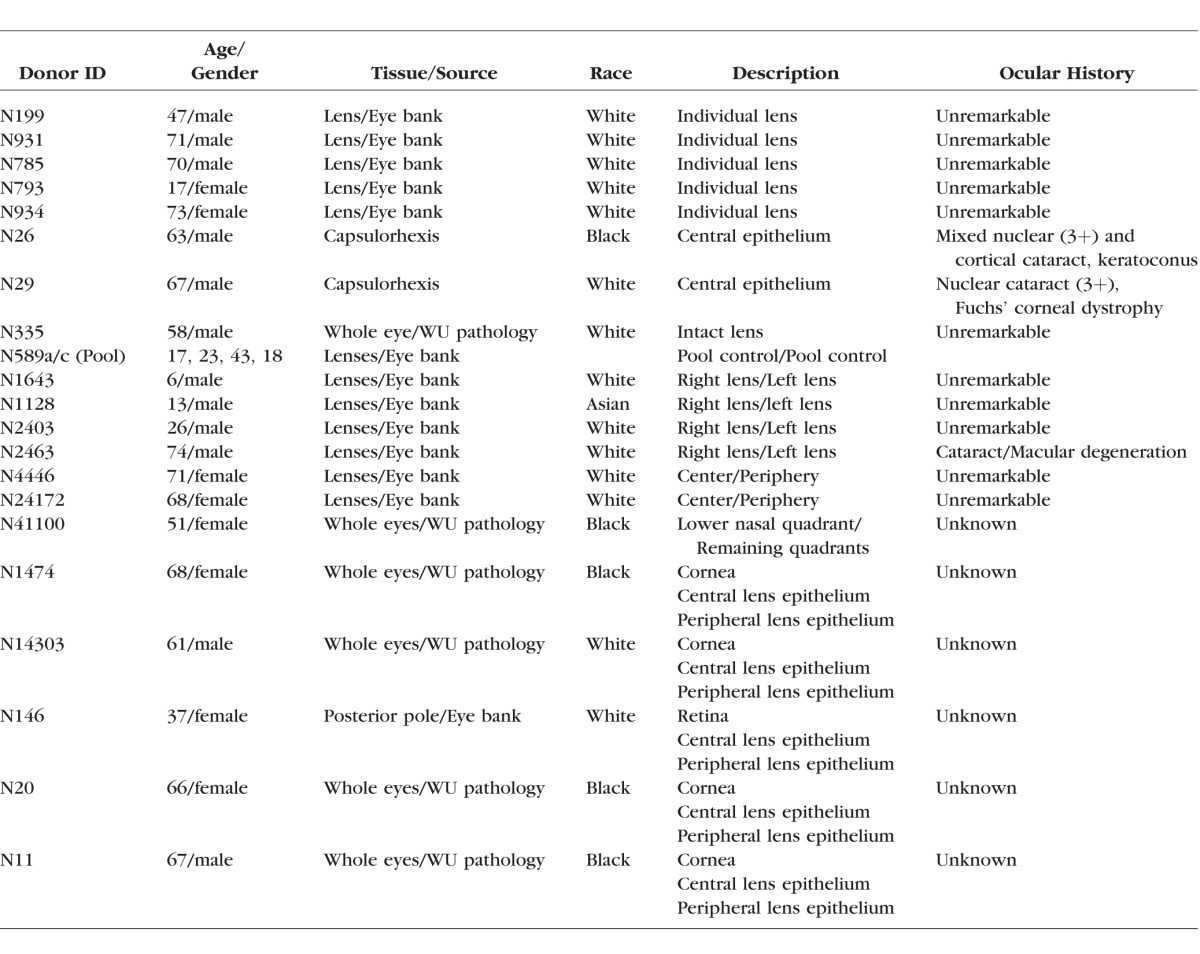
Description of Tissue Samples

### Dissection of the Lens Epithelium

**Figure 1:**
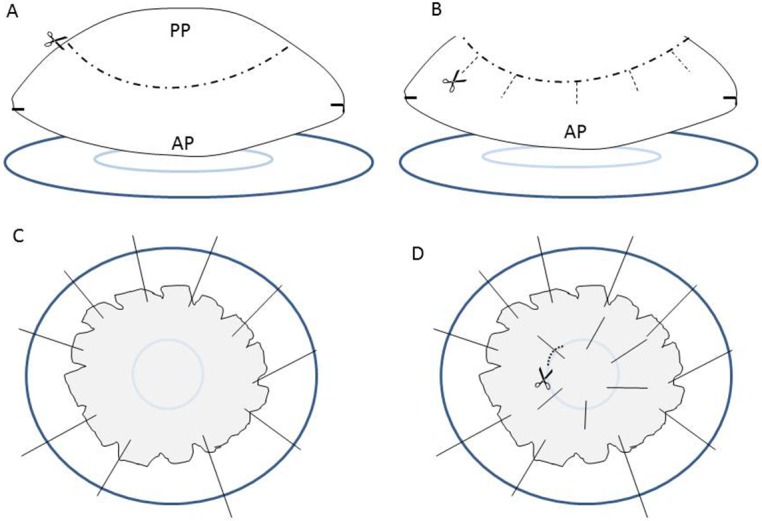
Dissection of the central and peripheral regions of the human lens epithelium. (**A**) The base of a 35-mm Petri dish (*dark blue*) is covered with four layers of Parafilm and imprinted with a 6-mm-diameter circle (*light blue*). The lens is positioned in the Petri dish with the anterior pole (AP) facing down. (**B**) A 7-mm-diameter circle is removed from the posterior pole (PP) and radial cuts are made in the remaining portion. (**C**) The lens is centered over the 6-mm circle, and the capsule is pinned to the base of the Petri dish bottom. The fiber cell mass is removed. (**D**) The central capsule (with its adherent layer of epithelial cells) is pinned to secure the tissue during dissection, and the central portion of the epithelium/capsule is removed using the 6-mm circle as a guide. The remaining part of the lens epithelium/capsule is collected separately.

The base of a 35-mm Petri dish was covered with four layers of Parafilm, and a 6-mm-diameter circle was imprinted into the surface by pressing the blunt end of a pipette tip into the Parafilm. The base of the dish was filled with sufficient PBS (NaCl 137 mM; KCl 2.7 mM; Na_2_HPO_4_ 10 mM; KH_2_PO_4_ 1.8 mM) to prevent dehydration of the lens tissue during dissection. Lenses were released from donor eyes by cutting the ciliary zonule. Lenses were transferred to the Petri dish and oriented such that the epithelium faced down. Using surgical scissors, a circular piece of the posterior capsule approximately 7 mm in diameter was removed and discarded (Fig. 1). A series of radial cuts was made in the remaining portion of the posterior capsule. Lenses were positioned over the marked circle in the Parafilm and the capsule pinned to the base of the dish. The fiber cell mass was gently removed and discarded. In some cases, the entire epithelium was used. In other cases, the central region of the epithelium, corresponding to the 6-mm circle on the Parafilm, was excised carefully and collected in a microfuge tube. The remaining epithelium, referred to here as the peripheral epithelium, was collected in a separate microfuge tube. In some samples, cells were harvested from that region of the epithelium corresponding to the lower nasal quadrant (LNQ). For that purpose, the original orientation of the eye in the head was determined from the external anatomy of the globe, and the orientation of the lens in the eye was monitored during dissection by making a small mark on the capsule, as described.^20^ The remaining quadrants (RQ) of the epithelium were collected in another tube.

### Library Preparation

DNA was isolated from fresh or frozen tissue using QIAamp DNA micro kit (QIAGEN, Inc., Valencia, CA, USA). Genomic DNA (0.2–1.0 μg) was fragmented by sonication (Covaris E210; Covaris, Inc., Woburn, MA, USA) to an average size of approximately 175 bp. DNA fragments were concentrated using AMPureXP beads (Beckman Coulter Genomics, Danvers, MA, USA), and DNA ends were repaired using T4 DNA polymerase, Klenow polymerase, and T4 polynucleotide kinase. The 3′ ends of the fragments were adenylated using exo-minus Klenow polymerase, allowing sequencing adapters to be ligated to the ends of the fragments. Adapter ligated DNA was subjected to limited (7–10 cycles) PCR amplification. Sequencing libraries were hybridized at 65°C for 24 hours to the Washington University Cancer Mutation Profiling version 2 (WUCaMP2) gene set^21^ using custom SureSelect biotinylated cRNA capture baits (Agilent Technologies, Santa Clara, CA, USA), according to the manufacturer's instructions. The WUCaMP2 panel includes coding exons from 151 cancer-related genes (see Table 2) totaling 0.83 Mb of captured sequence. The hybridized product was amplified for 14 PCR cycles using Agilent postcapture primers and a custom indexing primer.

**Table 2:**
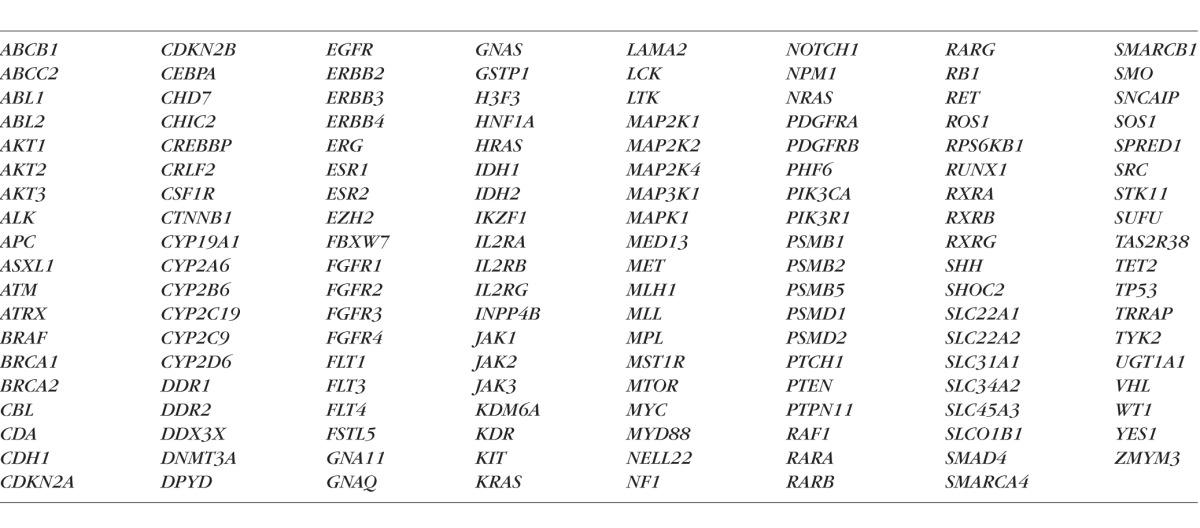
Genes in the WUCaMP2 Cancer Panel Used for Targeted NGS of Lens Epithelial Samples

### Sequencing and Base Calling

**Figure 2:**
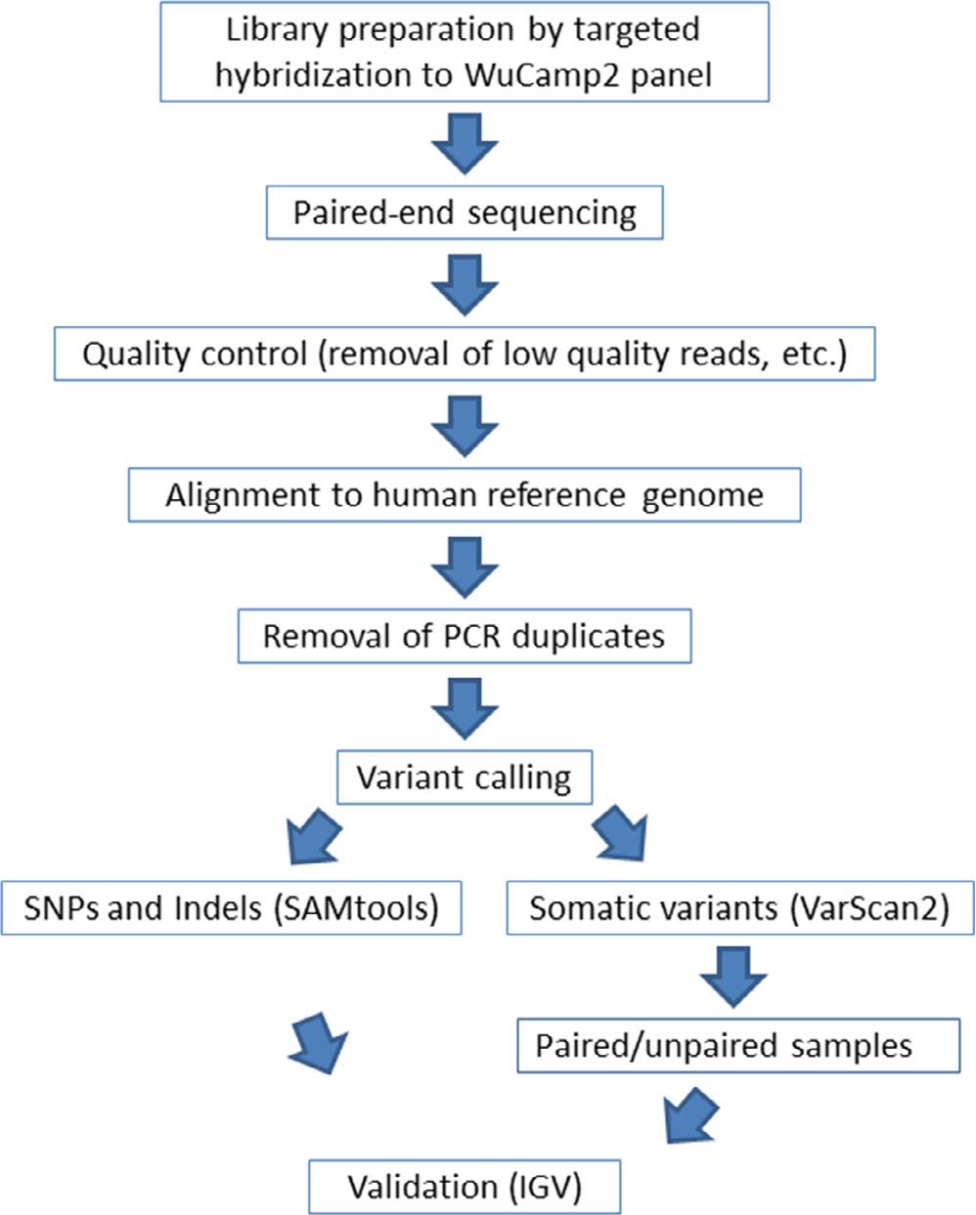
Work flow used to identify somatic variants in genomic DNA extracted from human lens epithelial cells. Sequencing libraries were enriched for genes of the WUCaMP2 panel (Table 2) by targeted hybridization capture. The resulting library was sequenced on an Illumina HiSeq 2500 platform to obtain paired-end reads. Sequencing results were filtered at several levels. The reads were then aligned to a human reference genome (hg19), and PCR duplicates were removed. Variants were called using SAMtools and VarScan2 software. Finally, selected variants were inspected manually using the IGV visualization tool.

Multiplex sequencing of the DNA libraries was performed on a HiSeq 2500 sequencing platform (Illumina Inc., San Diego, CA, USA) to obtain paired end 101-bp reads. Sequence reads were aligned to the human reference genome (hg19) using Novoalign (v. 3.02.06; Novocraft Technologies, Petaling Jaya, Malaysia). Polymerase chain reaction duplicates were removed from the alignments with Picard tools (v. 1.46; http://broadinstitute.github.io/picard/, available in the public domain), and variants were called using SAMtools (v. 0.1.18-1; https://github.com/samtools/samtools, available in the public domain). Variants were annotated with SnpEff software (http://snpeff.sourceforge.net/, available in the public domain).^22^ The mean coverages of the unique reads on target were obtained using Qualimap V2.1.1 (http://qualimap.bioinfo.cipf.es/, available in the public domain).^23^ The coverage of targeted bases was calculated using Bedtools (https://github.com/arq5x/bedtools2, available in the public domain).^24^ Genome visualization was performed using the Integrative Genome Browser data server.^25^ A schematic showing the sequencing and bioinformatic workflow is shown in Figure 2.

### Somatic Variant Calling

Somatic variants were identified using VarScan2 (http://varscan.sourceforge.net). To remove false positives that may arise through sequencing or alignment errors, a false-positive filter is incorporated into the VarScan2 algorithm.^26^ The filter evaluates putative variants according to nine empirically derived criteria designed to distinguish true somatic variants from artifactual calls. VarScan2 can operate in two modes: one for unpaired samples and the other for paired samples (somatic mode), where one of the two samples serves as a case and the other as a control.^27^ For single tissue samples (Table 1), VarScan2 was run in unpaired mode, using default parameters.^26^ To screen for putative somatic changes in unpaired samples, variants with frequencies lower than 40% were selected manually. From this group, statistically significant variants (*P* < 0.05; Fisher's Exact Test), covered to a depth greater than 300 with a minimum of four reads supporting the variant in both forward and reverse strands were evaluated further using the Integrative Genomics Viewer (IGV).

VarScan2 was used in somatic (paired) mode when matched case/control tissue pairs (for example lens/cornea or lens/retina) from a single donor were available (Table 1). Developed originally as a method for identifying rare variants in tumor samples, here we used VarScan2 to identify variants in lens samples that were absent from control tissues, allowing us to exclude single nucleotide polymorphisms (SNPs) or germline variants. The mpileup input files for VarScan2 were obtained from SAMtools mpileup v. 0.1.18 using default parameters, with Phred Base quality ≥20 and Phred Mapping quality ≥30. To call somatic variants, VarScan2 was run using the following parameters: Min coverage: 50x for Normal, 50x for “Tumor” (usually, lens); Min reads 2:2; Min strands 2:1; Min var freq: 0.01; Min freq for hom: 0.75; Normal purity: 1.0; Tumor purity: 1.0; Min avg qual: 15; *P* value thresh: 0.99; Somatic *P* value: 0.05, for all other settings, default parameters were used. Loss of heterozygosity (LOH) variants were taken as somatic variants. The following additional criteria were used to call a somatic variant: a minimum of 300 reads at the position of the variant for both case and control, a minimum of four reads supporting the variant present, in both forward and reverse strands, and the absence of the variant allele from the matched tissue. When a third specimen from the same donor was included as an extra control in the analysis (see Table 1), a minimum of 250 reads at the position of the variant for the case, a minimum of 300 reads at the position of the variant in the two controls, a minimum of four reads supporting the variant present in both forward and reverse strands, and the absence of the variant allele from the other two control specimens were required to score the variant as somatic. Finally, the reads supporting each putative somatic variant were evaluated manually using the IGV program. Variants found in reads with questionable mapping (more than three discrepancies), close to indels, or at the beginning or end of reads were discarded.^25,28^

### Droplet Digital PCR

In droplet digital PCR (ddPCR) a genomic DNA sample is partitioned into thousands of small droplets. A PCR reaction is performed within each droplet, using primers designed to differentiate between wild-type and variant sequences. The number of positive droplets is measured by fluorescence activated cell sorting (FACS) technology and the data are interpreted using Poisson statistics. The ddPCR technique was developed originally to quantify rare variants in the presence of a large background signal from the wild-type genome^29^ and variant allele frequencies as low as 1 in 200,000 have been measured successfully using the ddPCR approach.^30^ Here, we used ddPCR to validate independently one of the variants detected in the NGS analysis.

Digital droplet PCR was performed on a QX200 system (Bio-Rad Laboratories, Hercules, CA, USA) using TaqMan chemistry. Assays were designed as per MIQE guidelines.^31^ Primers and probes were used at final concentrations of 900 nM and 250 nM, respectively. Approximately 10 ng of genomic DNA was added to each reaction. After droplet generation, the droplets ( approximately 15,000 per reaction) were transferred to an Eppendorf TwinTec PCR 96-well plate. A temperature gradient (52° to 62°C) was used to determine optimum annealing temperature, using oligonucleotides for wild-type and variant sequences. The plate was run on a Bio-Rad C1000 thermal cycler: 95°C for 10 minutes, 95°C for 30 seconds, and 54.5°C for 1 minute × 39 cycles, 98°C for 10 minutes, and a 12°C hold. Droplets were read on a Bio-Rad QX200 droplet reader, and data were analyzed using QuantaSoft 1.7 (Bio-Rad). Thresholds were set manually, based on the location of clusters in the negative and positive controls. The negative control consisted of PCR reaction mix to which no DNA was added, whereas the positive control was a mixture of 10 ng/mL of genomic wild-type DNA and 2.5 × 10^−5^ ng of the variant synthetic DNA fragment. The variant was considered to be present in droplets with fluorescent signals of greater than 4000 in the FAM channel and less than 3000 in the HEX channel. The absolute copy number (copies/μL) of the variant allele (CN_VAR_) and the wild-type allele (CN_WT_) in each reaction was calculated from the Poisson distribution law^32^ and the variant allele fraction (VAF) was calculated as follows, VAF = CN_VAR_/(CN_VAR_ + CN_WT_).

## Results

### Isolation of Genomic DNA From Ocular Tissue Samples

We obtained lens epithelia from several sources, including surgical specimens and cadaveric tissue (Table 1). It was helpful to have two tissue samples from a single donor (so that somatic and germline variants could be readily differentiated), but depending on the source, this was not always possible. Another challenge was obtaining sufficient DNA for sequencing experiments. The lens epithelium contains approximately 0.5 × 10^6^ cells,^33^ and this limited number of cells did not always yield sufficient DNA for the analysis. This was particular true of the surgical (capsulorhexis) specimens, which constituted only a small (4–5-mm diameter) piece of the central lens epithelium. Nine pairs of capsulorhexis specimens had to be discarded for this reason. In postmortem samples, the lens cells did not always adhere tightly to the capsule, and this inevitably resulted in cell loss on dissection, with diminished yields of DNA as a consequence. Thus, only 50% of lens pairs, 33% of central/peripheral tissue pairs, and 25% of LNQ/RQ pairs provided sufficient high-quality DNA for sequencing. In total, 41 tissue samples from 24 donors/cataract patients were included in the study (Table 1).

### Sequencing Assessment

The mean total number of reads for each of the tissue samples examined in this study was 32 × 10^6^ (Supplementary Table S1). On average, 98% of the reads mapped to the reference sequence, and 97% were properly paired. An average of 56% of the reads mapped to the target region, implying high capture efficiency during library preparation. A high read depth was achieved across the target region. For example, after removal of PCR duplicates, 99% of the targeted bases were covered at ≥100-fold, 79% at ≥500-fold, and 42% at ≥1000-fold (Supplementary Table S2). Across all samples, the mean sequencing depth was 1018-fold (Supplementary Table S1).

To gauge the reproducibility of our sequencing/bioinformatics workflow, well-mixed DNA from a pooled sample of lens epithelial DNA (sample N598; Table 1) was divided into two aliquots (N589a and N589c), which were analyzed independently. The sequencing results, enrichment, alignment and coverage were comparable for N589a and N589c (Supplementary Table S1). In each sample, 269 known SNPs, 2 novel SNPs, and 12 Indels (insertions/deletions) were detected (Supplementary Table S3). Thus, at least for high-frequency variants (SNPs have a variant allele frequency of 50% or 100%, depending on whether they are heterozygous or homozygous), the sequencing technique was extremely reproducible.

### Variant Calling From Unpaired Samples

For eight of our samples, no control tissue was available (these comprised six lenses and two capsulorhexis specimens from eight individual donors; see Table 1). Sequence data obtained from these samples were, therefore, examined with VarScan2 in unpaired mode (Table 3). In the eight unpaired samples, 123 variants with frequencies of less than 25% were identified. This list was refined further by selecting variants covered to a depth of greater than 300 and supported by both forward and reverse strands. Of the 34 variants that met those conditions, 23 (67.5%) were detected in multiple samples (Table 3). Notably, 21 of the 23 variants were located in *CYP* family genes (*CYP2D6* and *CYP2A6*). Moreover, the variants at positions 41354661 and 41354606 were observed at low frequency in 35 of the 39 tissue samples sequenced in this study. It is unlikely that the same point substitution would occur independently in multiple samples, implying that the variants detected in the *CYP* family genes reflected sequencing or alignment errors. Manual inspection of the seven repetitive variants (Table 3) revealed that the paired end reads did not map to a single gene, but rather to two members of the *CYP* gene family located on the same chromosome. These imprecise alignments, a consequence of the high-sequence homology between *CYP* genes, were a source of false positives, and variants associated with these genes were therefore discarded. Similar observations were obtained for the unique variants (see Table 3) in *CYP2D6* (sample N26) and *CYP2A6* (sample N931). From the 34 variants identified initially, only nine (from samples N29, N793, and N934; Table 3) passed all quality control filters, with frequencies ranging from 1.02% to 3.94%. Of these, four were nonsynonymous missense substitutions and a fifth, in *ALK*, represented a splice site donor mutation.

**Table 3:**
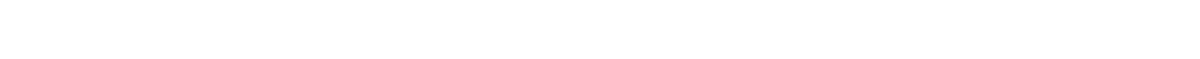
Somatic Variants Detected in Unpaired Lens Specimens

### Calling Somatic Variants From Paired Samples

VarScan2 was designed for calling low-frequency somatic variants from deep sequencing experiments on matched tissue samples. The algorithm, intended primarily for cancer studies, compares data from pairs of tissues (in our case, left versus right lens epithelia, lens versus cornea, or LNQ of the epithelium versus the RQs) obtained from a single individual. The software compares the number of reads containing the variant in each tissue sample and calculates a corresponding *P* value, using Fisher's Exact Test. Variants are classified as germline (if a variant is found in both control and case tissues), somatic (if the variant is found in the case only), or LOH (if the variant is found in the control tissue only). In our study, LOH variants were included under the somatic variant designation.

A total of 9622 putative variants (ranging from 615 to 1038 variants per sample) were detected in the 13 sets of two- and three-tissue comparisons (Table 4). Most of these were known SNPs or Indels, which are inherited through the germline and not considered further here. However, VarScan2 also identified 1001 low-allelic fraction variants, which were classified, provisionally, as somatic variants. Evaluation of the control DNA samples N589a and N589c (consisting of aliquots from a single pooled sample that were sequenced independently) with VarScan2 yielded 55 putative variants (Table 4), an indication of the potential false-positive discovery rate in the initial screen. Additional filters (described in Materials and Methods) were applied to further suppress the false-positive discovery rate. Following filtration, the number of variants in the control sample was reduced to zero. In the other 12 sets of tissue, 33 somatic variants passed all filters and are described in detail in Table 5.

**Table 4:**
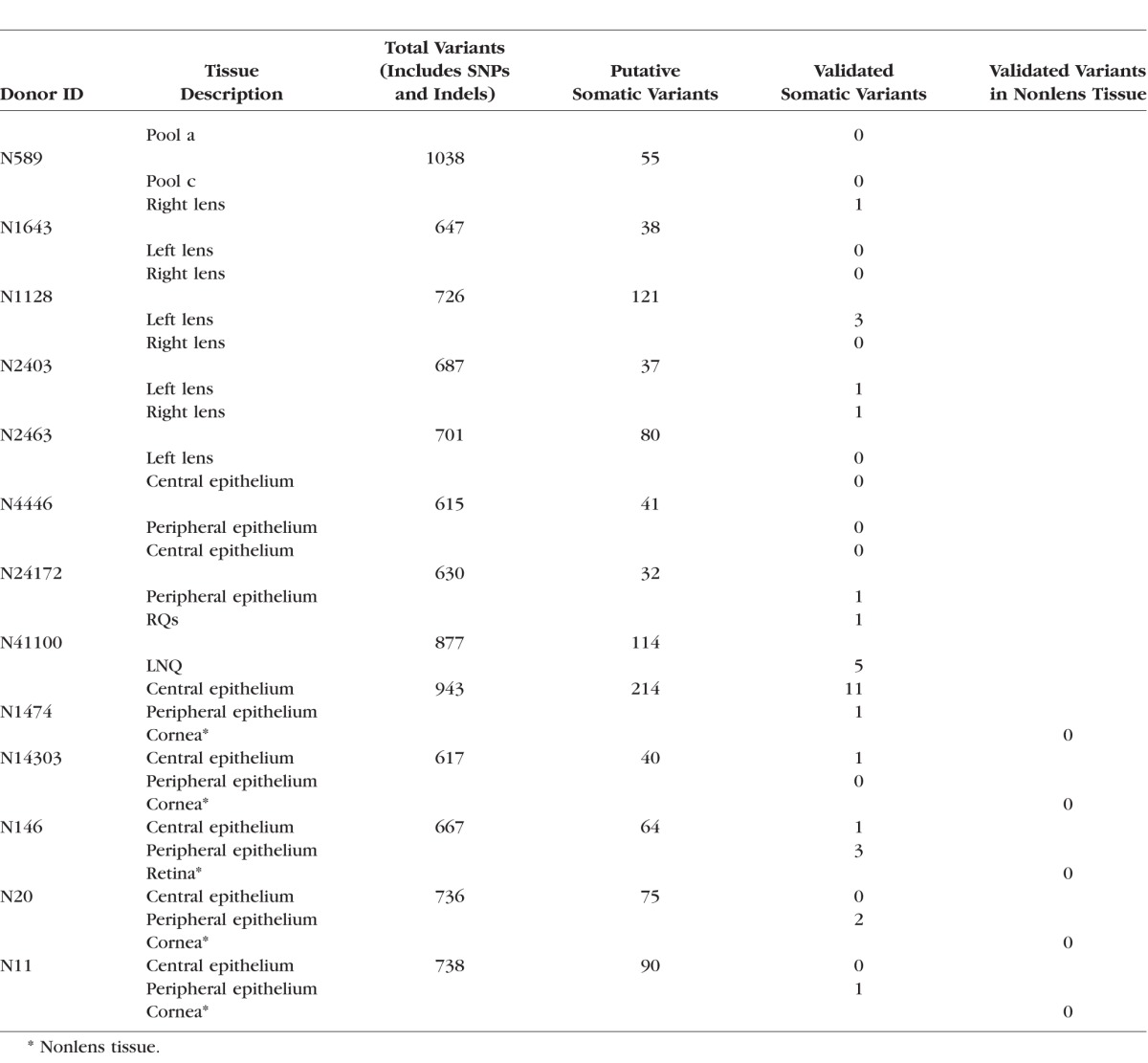
Variant Calling in Lens Specimens Based on Comparison With One or More Control Tissues

**Table 5:**
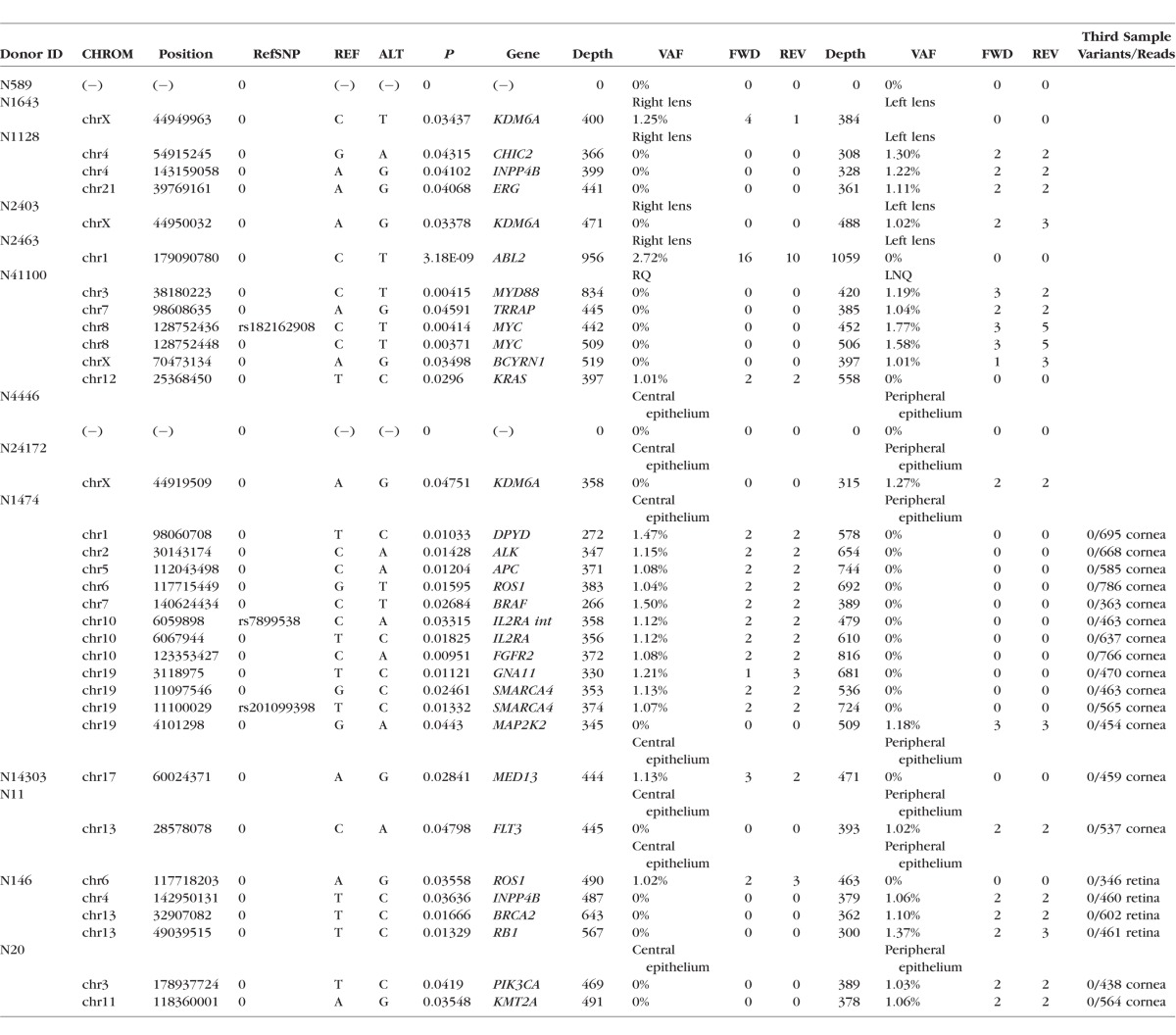
Detailed Annotation of Validated Somatic Variants Identified in Two- or Three-Way Tissue Comparisons

We examined the occurrence of somatic variants in four pairs of lens epithelia, obtained from donors (N1643, N1128, N2403, and N2463) ranging in age from 6 to 74 years (Table 5). Six somatic variants were identified in these samples, at frequencies of 1.02% to 2.72% (frequencies were derived from the number of reads supporting a variant/total number of reads). None of the variants were present in the SNP database and two were the result of C→T transitions at dipyrimidine sites, so-called UV signature mutations.

**Figure 3:**
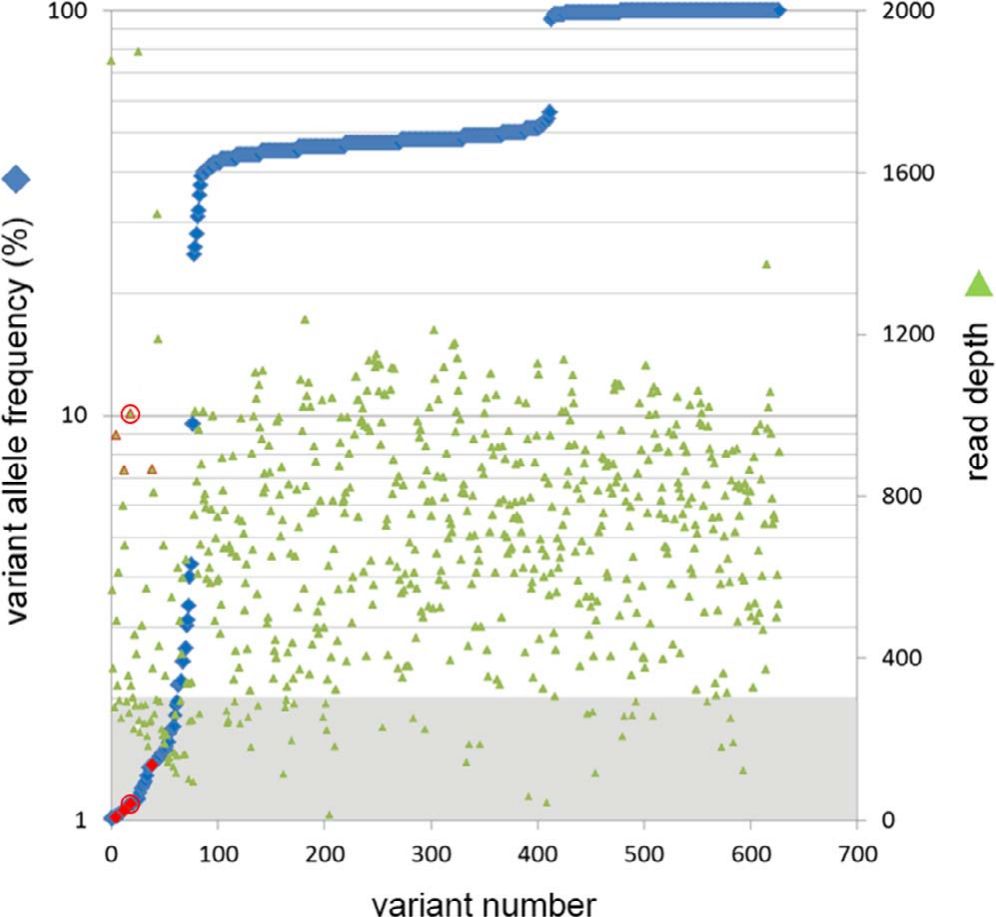
Variant allele frequency distribution in the peripheral lens epithelial sample from donor N146 (see Table 1). A total of 628 variants (*blue diamonds*) are detected with frequencies ranging from 1% to 100%. For each variant, the corresponding read depth is indicated (*green triangle*). Variants with frequencies of 50% or 100% represent heterozygous or homozygous SNPs, respectively. Seventy-eight low-allelic fraction (<10%) variants are detected. Many of the low-allelic fraction variants were not called as true somatic variants because they were associated with read depths of less than 300 (*shaded area*) or because they failed other quality control criteria (see Materials and Methods). Four variants (*red diamonds*) passed all filters (including sufficient read depth [*red triangles*]). The variant *circled in red* (the associated read depth value is also *encircled in red*) corresponds to a T > C substitution in the *BRCA2* gene (see Table 5).

Central lens epithelial cells are located directly on the optical axis of the eye and, therefore, exposed to sunlight. Conversely, cells located in the peripheral lens epithelium are situated in the shadow cast by the iris. We explored whether differential exposure to sunlight results in an uneven distribution of somatic variants across the epithelium. For these experiments, we extracted DNA from cells located in the sunlight-exposed central epithelium and compared it to DNA extracted from cells in the shaded peripheral epithelium (samples N4446, N24172, N1474, N14303, N11, N146, and N20). In some cases, a third tissue sample (cornea or retina) was included in the analysis (Table 5). An example of one such analysis is shown in Figure 3, and tabulated data for all the comparisons are provided in Table 5.

In central and peripheral samples, most (>90%) variants were present at frequencies of 50% or 100%, representing heterozygous or homozygous SNPs, respectively (see Fig. 3). However, some variants (identified using VarScan2) were present at markedly lower frequencies (1%–10%). Many of the low-allelic fraction variants were excluded from further analysis because they did not pass the quality control filters. Somatic variants were identified in six of the seven pairs of matched central/peripheral lens samples. In only one pair (N1474) were more somatic variants detected in the central epithelium than in the peripheral epithelium (Table 5). Thus, at least in this limited sample, there was no indication of a preponderance of somatic variants in the sunlight-exposed central region of the epithelium. Optical modeling of light exposure in the eye indicates that light exposure may not be equal at all radial locations and irradiance is predicted to be greatest in the LNQ of the lens epithelium.^34^ Using DNA from donor N41100, we compared the type and number of somatic variants in the LNQ with the remaining epithelial quadrants (RQ; Table 5). Five somatic variants were detected in the LNQ sample versus a single variant in the RQ sample.

**Figure 4:**
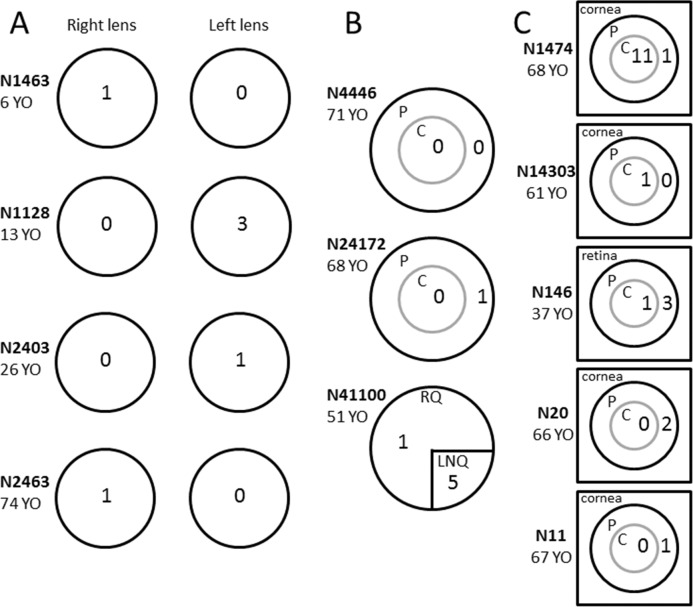
Somatic variants in matched ocular specimens. Tissue comparisons consisted of epithelia from right versus left lenses (**A**), central versus peripheral epithelium from individual lenses (**B**), or central versus peripheral lens epithelium versus cornea or retina (**C**). The number of somatic variants detected in each region is indicated. Additional information about the individual samples or the sequencing results is available in Tables 1, 4, and 5. P, peripheral epithelium; C, central epithelium; YO, years old.

In comparing sequence data from sets of matched ocular tissues obtained from 12 donors, 33 somatic variants were detected in 26 genes of the WUCaMP2 gene set. The spatial distribution of the variants is shown schematically in Figure 4.

### Droplet Digital PCR Analysis

**Figure 5:**
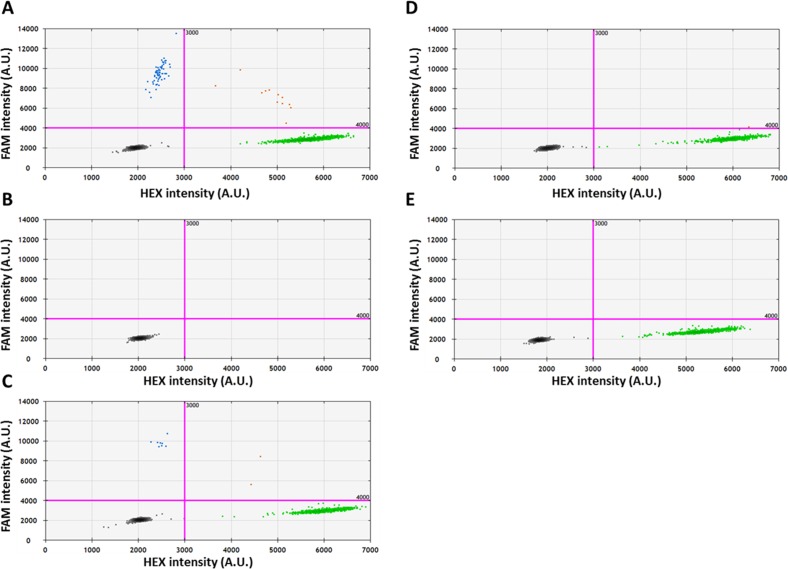
The presence of a *BRCA2* somatic variant detected in sample N146 (see Table 5; Fig. 3) is validated by ddPCR analysis. A TaqMan probe labeled with HEX fluorophore was designed to detect the wild-type (WT) *BRCA2* allele, and a probe labeled with FAM fluorophore was designed to detect the variant *BRCA2* sequence. The threshold lines (*pink*) were determined by the positive control (**A**), to separate clusters with WT or variant DNA. *Green dots* represent droplets with WT DNA, *blue dots* represent droplets with variant DNA, *brown dots* represent droplets with both WT and variant DNA, and *black dots* represent droplets with no target DNA. The variant was called as present in droplets with fluorescence units in FAM > 4000 and with fluorescence units in HEX < 3000. The DNA from the peripheral lens region (**C**) shows the presence of the variant, whereas samples from the central lens region (**D**) and from the retina (**E**) show the presence of WT DNA only. In the negative control (**B**), no HEX or FAM droplets were identified.

For most tissue samples used in this study, the amount of DNA was limiting and, consequently, the entire DNA sample was generally used for sequencing. In one case (N146), however, there was a small surplus of DNA. This was used to verify independently the existence of one of the somatic variants identified by deep sequencing and obtain an absolute measure of variant allele frequency by ddPCR analysis. Sequencing analysis of the tissue samples from donor N146 identified a somatic variant (T > C; position 32907082) in the *BRCA2* gene (Table 5) that was present in DNA extracted from the peripheral epithelium (red circle in Fig. 3) but absent from the central epithelium or the retina (Fig. 4C). Next generation sequencing suggested that the variant was present at a frequency of 1.10%. Primers were designed to differentiate the variant from the wild-type sequence, and ddPCR was performed on lens and retinal DNA from donor N146. Positive controls (consisting of wild-type or mutant DNA) were used to set threshold values, as described in Materials and Methods. In test samples, droplets meeting the threshold conditions were detected only in samples prepared from the peripheral lens epithelium and not from the central epithelium or retina (Fig. 5). In the peripheral lens epithelial sample, the variant allele frequency for the targeted *BRCA2* variant was 1.3%, close to the value of 1.1% obtained from the deep sequencing analysis (Table 5). Thus, using an orthogonal technique, ddPCR, we were able to substantiate the variant call from the deep sequencing data.

## Discussion

Epidemiologic studies have identified an elevated risk of developing cortical cataracts and PSCs after exposure to UV-B or ionizing radiation but the molecular mechanisms remain obscure.

The indeterminate threshold dose and increasing latency period between low-dose exposure and cataract formation suggests that, at least for radiation-induced opacities, the underlying process could involve a mutational component. It has been suggested that genotoxic damage may also contribute to the formation of common, age-related cataracts and, in that regard, it may be significant that micronuclei, biomarkers of DNA damage, are present in epithelial cells from cataractous lenses.^35^ Until recently, however, sequencing methods have lacked the sensitivity to determine directly whether somatic variants are present in the human lens.

Application of massively parallel sequencing technologies has begun to reveal the full burden of somatic mutations in untransformed mammalian cells. In postmitotic neurons, for example, each cell appears to have a distinct genome, harboring hundreds^36^ or perhaps thousands of single nucleotide variants.^37^ In sunlight-exposed tissues, such as skin, the mutational burden can be especially heavy, with a preponderance of C > T UV-induced signature mutations.^38^ In such a setting, mutations that confer a growth advantage are positively selected, leading to the emergence of subclonal lineages.

The lens is a unique tissue, containing populations of actively cycling cells, growth-arrested cells, and postmitotic cells. By virtue of its role in image formation, the lens is also subjected to lifelong exposure to light. Irradiance is not uniform, however, and cells located in the pupil space are exposed to considerably more light than those at the periphery. Irradiance may be highest of all in the LNQ of the epithelium.^34^ The current study was designed to assess whether somatic variants can be detected in the aging lens, the nature of the underlying sequence alterations, and the distribution of variants with respect to latitude and radial location in the epithelium (i.e., whether variants are more common in sunlight-exposed regions or regions harboring the proliferating cell populations).

In total, we detected 41 somatic variants in 151 genes from 29 human lens epithelial samples. The age of the donors varied from 6 to 74 years. In other model organisms, somatic variants accumulate with age, but interestingly, at different rates in different tissues.^39^ Variants arise through misincorporation of nucleotides during DNA replication or from improperly repaired lesions arising between replication cycles, so-called clock mutations.^40,41^ In our study, however, we were unable to confirm that variants are more common in samples from older patients, although such an association may be detected in a more comprehensive analysis. Similarly, we did not observe a consistent pattern in the distribution of variants across the lens epithelium; sometimes variants were concentrated in the central epithelium, and sometimes they were more numerous in the periphery. A single experiment on the angular distribution of variants within the epithelium revealed that variants were most common in the LNQ. This is an interesting observation because that region is exposed to more sunlight than the rest of the epithelium,^34^ and it is there that cortical cataracts often develop initially.^42^ It has been hypothesized that germinative zone cells in the LNQ may be particularly prone to UV damage through the phenomenon of peripheral light focusing.^43^ Clearly, more experiments will be required to substantiate the initial sequencing results.

Seven of the 41 somatic variants detected in the lens samples were C > T substitutions. Of these, four occurred at dipyrimidine sites and may, therefore, constitute UV-induced substitutions. It is instructive to contrast the mutational burden in the lens epithelium with that of cells in the nearby eyelid epidermis. In a recent study of 234 eyelid biopsies from four donors, 3760 somatic mutations were identified in a panel of 74 genes.^38^ This is a much higher mutational burden than seen in lens samples in the current study. Moreover, many of the mutations identified in skin were “driver” mutations (i.e., mutations that confer a growth advantage and thereby lead to clonal expansion) in important signaling pathways (notably, the Notch pathway), although the samples were nominally free from cancer. Significantly, most mutations in eyelid skin were C > T or CC > TT UV-signature mutations, thought to result from misrepaired CPDs.^13^ A higher burden of mutations might be expected in skin because the epidermal cells are exposed directly to sunlight. In contrast, to reach the lens epithelium, light has first to pass through the cornea. Although transmissivity of human cornea to visible light is high, experiments on enucleated eyes suggest that much of the UV-B is absorbed as it passes through the tissue,^44^ reducing the likelihood of CPD formation in the underlying lens cells.

The variant frequencies detected in the current study ranged from 1% to 4%, and it is pertinent to ask whether these values are reliable, and if so, what do they represent at the cellular level? The ability of massively parallel sequencing to identify somatic mutations is limited, ultimately, by the fidelity of the sequencing reactions^45^ and extremely rare variants are currently undiscoverable using this technology. For allelic fractions of 1% and higher, however, the VarScan2 program has been shown to reliably call variants, provided a sufficiently high sequence depth is achieved.^46^ We took all reasonable steps to exclude false positives (including sequencing the same pooled sample of DNA twice and manually inspecting the reads supporting every putative variant). In trying to be conservative, we probably excluded some valid variants, as noted by others.^26^ However, it is difficult to categorically exclude the possibility that some of the variants may have arisen from errors in sequencing or library preparation. On the single occasion we had sufficient DNA, we were able to use an orthogonal technique, ddPCR, to confirm independently the variant allele frequency of a *BRCA2* T > C mutation identified through NGS sequencing. Unfortunately, it is not practicable to devise custom ddPCR assays to independently validate each putative variant.

The presence of a variant at a frequency of approximately 1% (for example, the *BRCA2* T→C variant identified in sample N146 and confirmed by ddPCR) suggests that the variant was present in approximately 2% of the cells sampled (assuming that the variant allele was heterozygous). The primate lens epithelium contains approximately 0.5 × 10^6^ cells. The peripheral lens epithelial sample in which the *BRCA2* variant was detected constituted approximately half of the epithelial area, implying that the variant was present in approximately 5000 cells. The cell density of the peripheral human lens epithelium is approximately 5500 cells mm^−2^.^47^ The *BRCA2* mutant clone would therefore cover an area of approximately 1 mm^2^. Eyelid epidermis is modeled as a complex mosaic of somatic mutations nested in a clonal architecture, and large (millimeter-sized) clones are thought to result from the presence of driver mutations that confer a cellular growth advantage.^38^

Approximately half of the somatic variants detected in lens epithelial cells were nonsynonymous changes, with the potential to impact cell growth kinetics. As in the epidermis, the presence of such variants may confer a growth advantage that accounts for the emergence of large clones of cells. Alternatively, large clonally related cell clusters may form through the normal growth process of the lens. Lineage tracing studies in mouse lenses have shown that epithelial cells undergo multiple rounds of cell division as they traverse the germinative and pre-germinative zones, leading to the generation of clonal clusters numbering 30 to 50 cells.^48–50^ Although this is two orders of magnitude smaller than the predicted clone size in humans, we note that primate lenses contains many more cells than the mouse lens (0.5 × 10^6^ vs. 4.3 × 10^4^), and of course, humans have a significantly longer life span. It is likely that over the course of the human life span, lens epithelial cells undergo many more cell divisions than mouse lens cells. As a consequence, it is plausible that somatic variants arising early in a lens epithelial cell lineage may reach frequencies of greater than 1% later in life.

In the current study, we screened a panel of genes (WUCaMP2) implicated in cancer for the presence of somatic variants. Although some of these genes may have relevance for cataract development, there is no reason to suspect, a priori, that sequence variations in these particular genes contribute directly to lens cell opacification. They were chosen primarily because they have been validated for use in hybridization capture analysis.^21^ However, a set of genes with essential roles in the maintenance of lens transparency has been identified through studies of inherited forms of cataract.^51^ Many of these genes encode proteins that are strongly or specifically expressed in lens tissue, such as α-, β-, or γ-crystallin, and gap junction proteins, such as connexin46 and connexin50. In brain tissue, single nucleotide variants are most common in actively transcribed genes, suggesting that mutations are introduced during transcription rather than DNA replication.^37^ If this is also true in the lens, then genes encoding crystallins and other strongly expressed proteins may be particularly prone to somatic mutation. The Cat-Map, an up-to-date listing of genes implicated in Mendelian or age-related forms of cataract, now includes more than 200 genes distributed across all 22 autosomes and the X-chromosome.^52^ Future sequencing efforts will focus on this set of genes with demonstrated roles in the maintenance of lens fiber cell transparency. If somatic variants can be identified in Cat-Map genes, it will strengthen the hypothesis that somatic mutations may play a contributory role in human cataract formation.

## Supplementary Material

Supplement 1

Supplement 2

Supplement 3
