# Supplementary material for: Somatic Variants in the Human Lens Epithelium: A Preliminary Assessment
Source: Invest Ophthalmol Vis Sci. 2016 Aug 8;57(10):4063–75. doi: 10.1167/iovs.16-19726 (PMC4986767; doi:10.1167/iovs.16-19726)
Supplement: Supplement 1 [file i1552-5783-57-10-4063-s01.pdf]

	Donor ID	Specimen type	Total reads	Reads mapped	Proportion of reads mapped	Properly paired reads	Mapped reads on target (%)	Reads remaining after removal of duplicates (%)	Unique reads on target	Mean coverage	S.D.
**Unpaired specimens**	**N29**	capsulorhexis	43,062,364	42,284,701	98.2%	95.74%	49.8	57.0	12,007,196.92	1,406	681
	**N26**	capsulorhexis	43,864,352	43,161,796	98.4%	96.0%	51.0	51.8	11,571,528.54	1,307	567
	**N931**	lens	35,984,830	35,417,590	98.4%	96.3%	55.5	59.5	11,871,209.33	1,437	797
	**N785**	lens	40,988,756	40,029,845	97.7%	95.4%	38.9	74.7	11,923,746.99	1,381	800
	**N199**	lens	40,262,396	39,588,675	98.3%	96.0%	53.4	64.8	13,945,129.68	1,710	982
	**N793**	lens	42,481,436	41,834,491	98.5%	96.3%	54.6	53.5	12,405,427.11	1,450	745
	**N335**	lens	38,242,982	37,465,891	98.0%	95.7%	51.2	68.5	13,414,151.10	1,622	943
	**N934**	lens	38,695,586	38,014,957	98.2%	95.9%	51.1	62.2	12,298,527.92	1,479	820
**Paired specimens**	**N589**	pool c	20,186,978	19,871,168	98.4%	94.5%	61.6	63.7	7,913,739.42	606	190
		pool a	29,782,448	20,541,313	98.8%	97.4%	60.2	67.8	12,154,256.93	697	236
	**N1643**	R lens	23,173,160	22,101,151	95.4%	90.4%	58.4	79.3	10,731,039.73	807	300
		L lens	24,988,528	24,335,350	97.4%	94.2%	60.1	71.1	10,676,544.05	830	296
	**N1128**	R lens	22,990,078	22,700,272	98.7%	97.4%	60.8	60.3	8,416,679.11	620	224
		L lens	17,837,412	17,185,641	96.4%	92.7%	65.0	76.1	8,823,425.99	700	304
	**N2403**	R lens	21,800,044	21,379,277	98.1%	96.0%	60.3	69.0	9,064,886.31	737	255
		L lens	23,571,208	23,330,143	99.0%	97.7%	61.9	69.4	10,122,980.28	871	315
	**N1474**	peripheral epithelium	22,072,418	21,797,848	98.8%	97.1%	65.2	62.4	8,979,908.60	677	365
		cornea	25,702,950	25,398,261	98.8%	97.1%	58.2	62.4	9,322,760.20	496	135
		central epithelium	20,281,514	20,061,387	98.9%	97.0%	63.6	49.4	6,374,211.07	375	107
	**N41100**	lower nasal quadrant (LNQ)	21,456,582	21,117,346	98.4%	97.7%	59.5	68.4	8,734,709.47	676	635
		remaining quadrants (RQ)	20,661,138	20,437,348	98.9%	97.3%	62.5	55.8	7,198,583.51	496	136
	**N2463**	R lens	14,265,620	13,840,704	97.0%	93.9%	60.7	77.1	6,671,216.37	546	209
		L lens	20,914,808	20,264,636	98.9%	93.7%	61.0	67.2	8,575,693.05	642	313
	**N4446**	central epithelium	42,103,332	41,587,902	98.8%	97.9%	53.7	64.2	14,531,322.37	1,716	1,000
		peripheral epithelium	39,725,084	39,297,952	98.9%	97.4%	52.3	62.5	12,989,575.57	1,467	841
	**N14303**	peripheral epithelium	36,509,356	35,949,238	98.5%	97.8%	45.8	74.9	12,530,757.44	1,523	962
		central epithelium	38,394,816	37,660,197	98.1%	97.3%	42.6	76.3	12,493,091.05	1,530	957
		cornea	38,865,382	32,563,868	98.4%	97.3%	42.6	77.2	12,780,493.05	1,506	862
	**N24172**	central epithelium	48,438,574	47,790,165	98.7%	97.4%	51.2	63.7	15,802,107.94	1,716	914
		peripheral epithelium	32,978,218	32,237,196	98.7%	97.8%	51.8	72.6	12,399,924.07	1,571	889
	**N146**	retina	36,548,862	35,946,699	98.4%	97.0%	51.8	51.3	9,713,070.27	455	104
		peripheral epithelium	35,553,918	35,256,604	99.2%	98.8%	70.7	44.8	11,268,528.89	836	385
		central epithelium	38,006,088	37,363,602	98.3%	97.7%	49.1	63.1	11,768,947.54	772	252
	**N20**	cornea	36,917,002	36,410,907	98.6%	97.5%	55.2	60.3	12,279,017.68	808	472
		peripheral epithelium	37,119,832	36,511,965	98.4%	97.9%	50.3	64.8	12,105,411.34	833	342
		central epithelium	34,454,328	34,050,520	98.8%	98.3%	61.1	57.3	12,070,360.04	903	339
	**N11**	cornea	35,900,498	35,491,875	98.9%	98.1%	61.9	57.9	12,860,881.98	988	367
		peripheral epithelium	38,188,958	37,804,220	99.0%	98.7%	66.3	43.2	10,924,769.36	655	243
		central epithelium	34,891,502	34,521,823	98.9%	98.5%	63.8	53.2	11,831,129.46	847	311
**mean total n=39**			**32,022,137**	**31,348,834**	**98.4%**	**96.6%**	**56.3**	**63.6**	**11,065,306.15**	**1,018**	**502**

Supplementary Table1: Alignment Quality
