# Supplementary material for: Somatic Variants in the Human Lens Epithelium: A Preliminary Assessment
Source: Invest Ophthalmol Vis Sci. 2016 Aug 8;57(10):4063–75. doi: 10.1167/iovs.16-19726 (PMC4986767; doi:10.1167/iovs.16-19726)
Supplement: Supplement 2 [file i1552-5783-57-10-4063-s02.pdf]

	Donor	Specimen	≥100	≥300	≥400	≥500	≥800	≥1000
**Unpaired specimens**	**N29**	capsulorhexis	0.99	0.98	0.96	0.93	0.80	0.69
	**N26**	capsulorhexis	1.00	0.98	0.98	0.93	0.80	0.68
	**N931**	individual lens	0.99	0.97	0.97	0.91	0.79	0.65
	**N785**	individual lens	0.99	0.98	0.93	0.89	0.73	0.63
	**N199**	individual lens	1.00	0.99	0.96	0.94	0.83	0.74
	**N793**	individual lens	0.99	0.97	0.96	0.94	0.78	0.68
	**N335**	individual lens	0.99	0.97	0.95	0.93	0.81	0.71
	**N934**	individual lens	1.00	0.99	0.98	0.93	0.78	0.68
**Paired specimens**	**N589**	pool c	0.99	0.94	0.86	0.72	0.19	0.00
		pool a	0.99	0.98	0.90	0.78	0.37	0.10
	**N1643**	R lens	0.99	0.98	0.89	0.81	0.50	0.30
		L lens	0.99	0.96	0.92	0.86	0.55	0.30
	**N1128**	R lens	0.99	0.90	0.83	0.70	0.23	0.50
		L lens	0.99	0.91	0.82	0.73	0.38	0.20
	**N2403**	R lens	0.99	0.95	0.90	0.82	0.43	0.18
		L lens	1.00	0.97	0.92	0.86	0.59	0.35
	**N2463**	R lens	0.99	0.87	0.74	0.58	0.13	0.02
		L lens	0.99	0.93	0.86	0.60	0.25	0.40
	**N4446**	central epithelium	1.00	0.95	0.93	0.90	0.78	0.71
		peripheral epithelium	0.98	0.93	0.90	0.87	0.75	0.67
	**N24172**	central epithelium	1.00	0.97	0.95	0.92	0.82	0.73
		peripheral epithelium	0.99	0.96	0.93	0.90	0.77	0.50
	**N41100**	lower nasal quadrant LNQ)	0.95	0.82	0.75	0.66	0.38	0.23
		remaining quadrants (RQ)	0.99	0.90	0.77	0.53	0.00	0.00
	**N1474**	peripheral epithelium	0.99	0.93	0.88	0.78	0.38	0.15
		cornea	1.00	0.96	0.92	0.84	0.36	0.10
		central epithelium	0.99	0.78	0.50	0.10	0.00	0.00
	**N14303**	peripheral epithelium	0.98	0.91	0.88	0.85	0.73	0.64
		central epithelium	0.99	0.95	0.90	0.87	0.74	0.65
		cornea	0.99	0.95	0.92	0.88	0.76	0.67
	**N146**	retina	1.00	0.93	0.75	0.36	0.00	0.00
		peripheral epithelium	0.99	0.95	0.92	0.86	0.63	0.43
		central epithelium	1.00	0.96	0.92	0.86	0.50	0.20
	**N20**	cornea	0.93	0.80	0.74	0.68	0.60	0.39
		peripheral epithelium	0.98	0.93	0.88	0.82	0.55	0.35
		central epithelium	0.99	0.95	0.90	0.87	0.74	0.64
	**N11**	cornea	1.00	0.97	0.93	0.88	0.69	0.56
		peripheral epithelium	0.98	0.90	0.84	0.74	0.32	0.50
		central epithelium	0.99	0.95	0.90	0.85	0.60	0.36
		**Mean**	0.99	0.94	0.88	0.79	0.54	0.42
		**S.D.**	0.01	0.05	0.09	0.17	0.26	0.25

Supplementary Table 2: Targeted base coverage
