# Supplementary material for: Somatic Variants in the Human Lens Epithelium: A Preliminary Assessment
Source: Invest Ophthalmol Vis Sci. 2016 Aug 8;57(10):4063–75. doi: 10.1167/iovs.16-19726 (PMC4986767; doi:10.1167/iovs.16-19726)
Supplement: Supplement 3 [file i1552-5783-57-10-4063-s03.pdf]

	Donor	Specimen	SNPs	novel SNPs	indels
**Unpaired specimens**	**N29**	capsulorhexis	210	10	9
	**N26**	capsulorhexis	268	19	13
	**N931**	individual lens	194	3	8
	**N785**	individual lens	178	1	8
	**N199**	individual lens	192	1	7
	**N793**	individual lens	198	2	8
	**N335**	individual lens	182	2	9
	**N934**	individual lens	197	3	9
**Paired specimens**	**N589**	pool c	269	2	12
		pool a	269	2	12
	**N1643**	R lens	267	1	11
		L lens	267	1	11
	**N1128**	R lens	268	8	11
		L lens	268	8	11
	**N2403**	R lens	253	6	13
		L lens	253	6	13
	**N2463**	R lens	265	10	22
		L lens	265	10	22
	**N4446**	central epithelium	200	2	10
		peripheral epithelium	200	2	10
	**N24172**	central epithelium	204	1	11
		peripheral epithelium	204	1	11
	**N41100**	lower nasal quadrant (LNQ)	225	10	18
		remaining quadrants (RQ)	225	10	18
	**N1474**	central epithelium	237	15	24
		peripheral epithelium	237	15	24
		cornea	237	15	24
	**N14303**	central epithelium	193	1	10
		peripheral epithelium	193	1	10
		cornea	193	1	10
	**N146**	central epithelium	224	9	19
		peripheral epithelium	224	9	19
		retina	224	9	19
	**N20**	central epithelium	246	10	17
		peripheral epithelium	246	10	17
		cornea	246	10	17
	**N11**	central epithelium	242	8	14
		peripheral epithelium	242	8	14
		cornea	242	8	14

Supplementary Table 3: Summary of SNPs and Indels
